# Narrow genetic base shapes population structure and linkage disequilibrium in an industrial oilseed crop, *Brassica carinata* A. Braun

**DOI:** 10.1038/s41598-020-69255-w

**Published:** 2020-07-28

**Authors:** Yogendra Khedikar, Wayne E. Clarke, Lifeng Chen, Erin E. Higgins, Sateesh Kagale, Chu Shin Koh, Rick Bennett, Isobel A. P. Parkin

**Affiliations:** 1 Agriculture and Agri-Food Canada, 107 Science Place, Saskatoon, SK Canada; 2 National Research Council Canada, 110 Gymnasium Place, Saskatoon, SK Canada; 3 Agrisoma Biosciences Inc., 110 Gymnasium Place, Saskatoon, SK Canada; 4 Global Institute of Food Security, Saskatoon, SK Canada

**Keywords:** Natural variation in plants, Plant genetics

## Abstract

Ethiopian mustard (*Brassica carinata* A. Braun) is an emerging sustainable source of vegetable oil, in particular for the biofuel industry. The present study exploited genome assemblies of the Brassica diploids, *Brassica nigra* and *Brassica oleracea,* to discover over 10,000 genome-wide SNPs using genotype by sequencing of 620 *B. carinata* lines. The analyses revealed a SNP frequency of one every 91.7 kb, a heterozygosity level of 0.30, nucleotide diversity levels of 1.31 × 10^−05^, and the first five principal components captured only 13% molecular variation, indicating low levels of genetic diversity among the *B. carinata* collection. Genome bias was observed, with greater SNP density found on the B subgenome. The 620 lines clustered into two distinct sub-populations (SP1 and SP2) with the majority of accessions (88%) clustered in SP1 with those from Ethiopia, the presumed centre of origin. SP2 was distinguished by a collection of breeding lines, implicating targeted selection in creating population structure. Two selective sweep regions on B3 and B8 were detected, which harbour genes involved in fatty acid and aliphatic glucosinolate biosynthesis, respectively. The assessment of genetic diversity, population structure, and LD in the global *B. carinata* collection provides critical information to assist future crop improvement.

## Introduction

Ethiopian mustard, *Brassica carinata* A. Braun, has been proposed as an industrial oilseed crop owing to its fatty acid profile with high levels of long and very long chain unsaturated fatty acids^1,2^. It is an allotetraploid formed through interspecific hybridization between ancestors of diploid *Brassica nigra* (B genome; 2n = 16) and *Brassica oleracea* (C genome; 2n = 18)^3^, with 2n = 4x = 34 chromosomes and a genome size of ~ 1,284 Mbp^4^. It is believed to have originated in the highlands of Ethiopia, and possibly adjoining parts of East Africa and the Mediterranean coast^5^. The crop is gaining importance in Western Canada, Southern Europe, Australia, South America and India because of its drought tolerance^6^, shatter resistance, large seed size^7^, and high level of resistance to blackleg^8^, and Alternaria leaf spot^9^. Although the oil is considered to be of lower nutritional value due to high levels of erucic acid (> 40%) it is being considered as an alternative source of biofuel feedstock, for example in the production of jet biofuel^2,10^.

There were limited genomic resources available that can be used for *B. carinata* crop improvement and low levels of molecular variation were identified using RAPD and AFLP technology for a relatively small number of lines^11,12^. However, progress has been made recently in the construction of genetic maps for the crop and quantitative trait locus (QTL) mapping using largely anonymous marker systems. The first linkage map was constructed using 212 SSR loci by Guo et al.^13^, while more recently 4,031 DArTseq loci were integrated into a map of the 17 chromosomes of *B. carinata*^14^. In addition, a gene conferring resistance to black rot was mapped to B7 using intron length polymorphism markers^15^. More recently, a diversity panel of 83 *B. carinata* accessions were evaluated using DArTseq, and QTLs associated with pod shatter resistance were mapped using an F_2_ population^16^. In addition, an independent panel of 81 accessions of *B. carinata* was genotyped using DArTseq to study population structure, and pattern of linkage disequilibrium and QTLs accounting for agronomic and seed quality traits were mapped using a doubled haploid (DH) mapping population^17^.

With the rapid development of next-generation sequencing platforms single nucleotide polymorphisms (SNPs) have become the marker system of choice in plant genetic studies, particularly in the construction of high-density linkage maps, QTL mapping, association analysis, and genetic diversity studies^18^. Exploiting high throughput sequencing platforms in approaches such as genotyping by sequencing (GBS) has enabled large numbers of accessions to be genotyped in a relatively cost-effective fashion^19,20^. The GBS assay utilizes restriction enzyme digestion to reduce the genome area being sequenced, creating increased coverage for target sites, and the use of methylation sensitive enzymes limits the capture of repetitive DNA regions^20^. Although there are some limitations, the modified GBS method has been successfully implemented in multiple diploid and polyploid species^20–23^.

The nucleotide diversity of crop plants is influenced by both natural and artificial selection^24^, thus characterisation of genetic diversity and population structure can unravel the evolutionary history and assist in maintaining and exploiting the available variation for a species*.* In addition, the characterised genetic variation can be employed in genetic mapping and genome-wide association studies (GWAS) for target trait analyses. Genetic analyses can also allow the calculation of local and genome-wide linkage disequilibrium (LD), the non-random association between alleles at loci across the genome, which is important in mapping studies as it dictates the resolution with which a trait can be determined^25^. Furthermore, haplotype analysis and selective sweep analyses can identify signatures of natural and artificial selection^26,27^. GWAS has proven to be a powerful tool to locate important genes underlying complex phenotypic traits in animal and plant studies^28,29^; however, it can be challenging in polyploid species such as those of the *Brassicaceae* due to the complexity and underlying redundancy of the genome.

Using available genome sequences for the Brassica diploids, *B. nigra* and *B. oleracea*, as a pseudo-reference for the *B. carinata* genome, the configuration of nucleotide polymorphism in *B. carinata* was explored. A worldwide collection of 620 lines was assayed, with thousands of high confidence SNPs distributed on the 17 chromosomes of *B. carinata*. These data revealed levels of genetic diversity, linkage disequilibrium, and haplotype patterns across the genome and identified genomic regions showing evidence of selective pressure. Conventional plant breeding has had limited success in *B. carinata* improvement^30^; however, the application of modern strategies such as marker assisted breeding has the potential to accelerate further development of this underutilised crop and the information and resources presented here should enable these goals.

## Results

### Single nucleotide polymorphism (SNP) discovery

**Figure 1:**
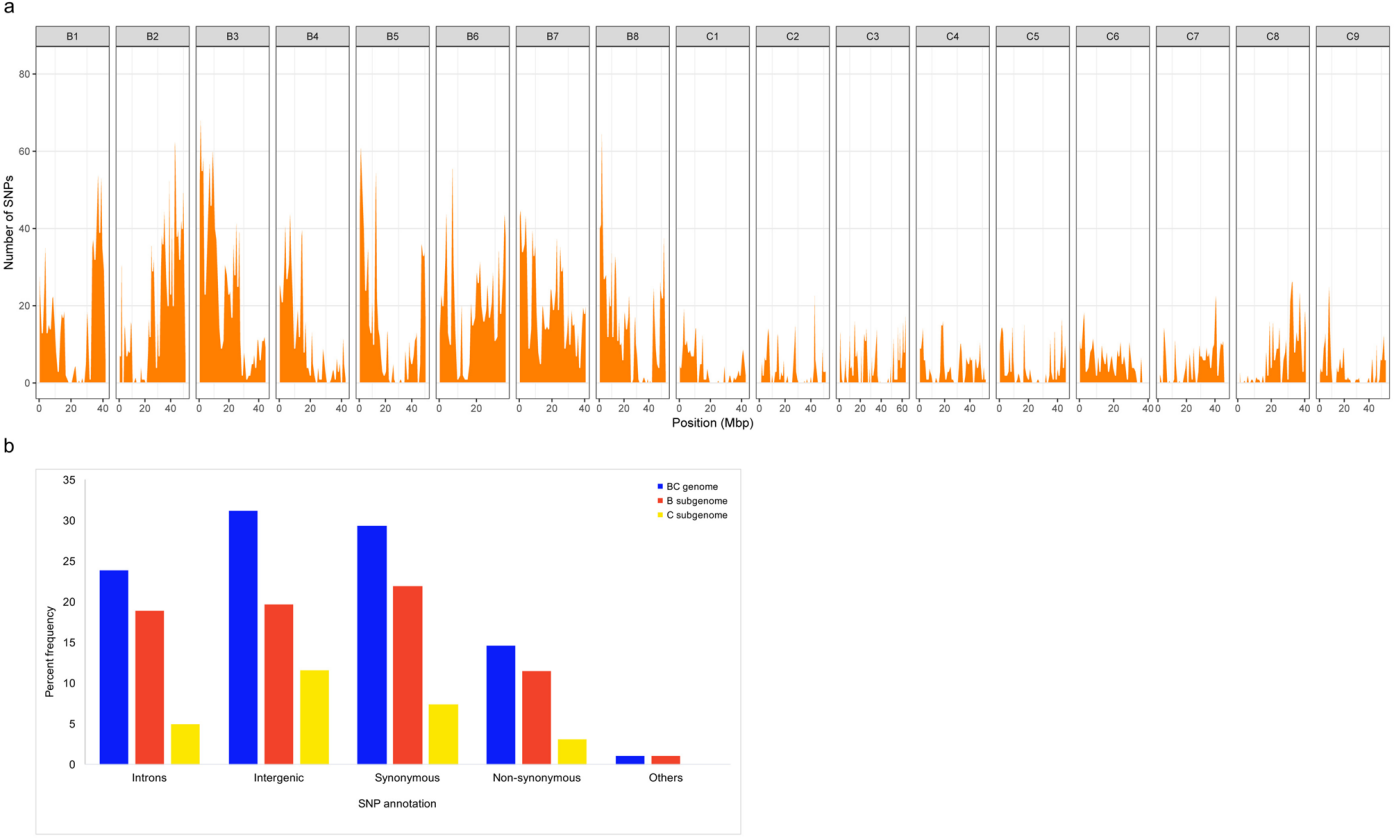
(**a**) Distribution of genome-wide SNPs across B and C subgenomes in 1 MB window. (**b**) The annotation of SNPs.

Genotyping by sequencing (GBS) of a global collection of 620 *B. carinata* accessions allowed detection of genome-wide SNP loci. For each accession, genome complexity reduction was carried out using two enzymes, *PstI* and *MspI,* followed by 96-fold multiplexed sequencing on an Illumina Hiseq2000, which yielded a total of ~ 660 million paired-end reads. The reads were aligned to a pseudo-reference genome for *B. carinata* composed of the concatenated genomes of the diploids *B. nigra*^31^ (https://Cruciferseq.ca) and *B. oleracea*^32^ (Supplementary Table S1a). The read mapping efficiency ranged from 66 to 94%, with an average of 84% of the reads uniquely mapping to the pseudo-reference. A total of 536,496 raw SNPs and indels were identified among the *B. carinata* collection. Of these, 10,199 high-quality SNPs were selected for genetic diversity and LD analysis based on a minor allele frequency (MAF) > 0.05, heterozygosity ≤ 0.1, read depth ≥ 4 (per line), and up to 30% missing data, at any locus (Fig. 1a). The high confidence SNP loci provided an average density of one SNP every 91.7 kb in the *B. carinata* pseudo-genome, with a higher prevalence of SNPs being detected in the Brassica B genome (one every 59.9 kb) compared to the C genome (one every 177.9 kb). The majority (43.9%) of the 10,199 high confidence SNPs were in coding regions, followed by intergenic regions (31.2%) and introns (23.9%) (Supplementary Table S2, Fig. 1b). Of the SNPs in coding regions, 66% were synonymous, and 33% were non-synonymous (Supplementary Table S2).

### Population structure

**Figure 2:**
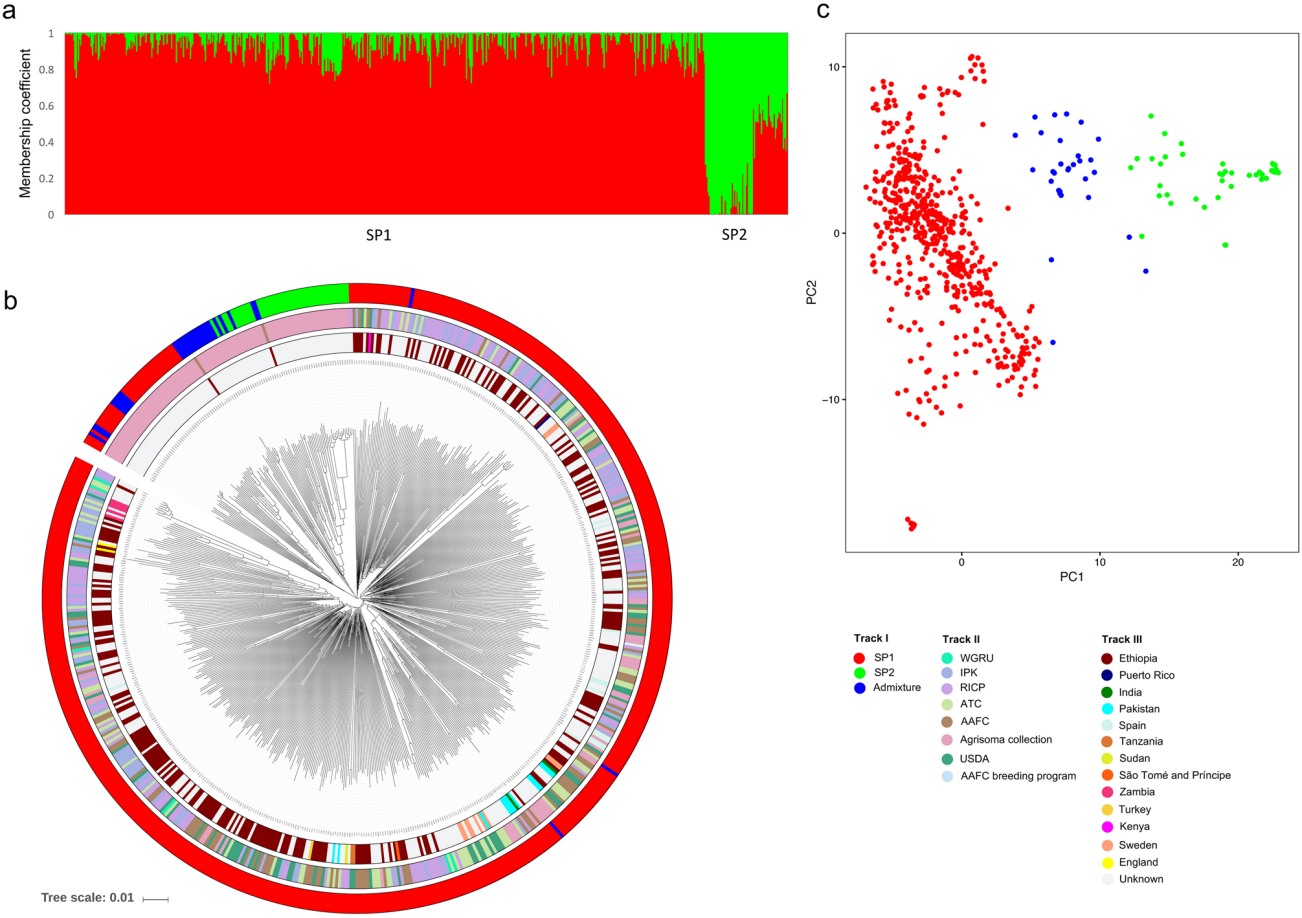
Summary of population analysis for worldwide *B. carinata* collection (**a**) Bar chart of inferred population structure for K = 2 from STRUCTURE. (**b**) Phylogenetic analysis; track I indicates subpopulations identified by STRUCTURE (SP1 are coloured in red; SP2 are green and AG are blue); track II shows the source of accessions (Gene banks); track III indicate the country of origin. (**c**) Principal component analysis (PCA) of 620 accessions.

STRUCTURE analyses using 1,535 unlinked (*r*^2^ = 0.1) genome-wide SNPs revealed two subpopulations (∆K value was highest at K = 2) among the 620 *B. carinata* accessions**.** Using a membership probability threshold of 70%, 549 (88.5%) accessions were assigned to subpopulation 1 (SP1), 41 (6.6%) to subpopulation 2 (SP2), and the remaining 30 (4.8%) were retained in the admixture group (AG) (Fig. 2a). SP1 comprised accessions from Ethiopia (99% of the total Ethiopian accessions) and accessions from a collection provided by Agrisoma (53% of the Agrisoma collection). In addition, a small number of accessions (7% of the total) purporting to originate from a diverse range of countries (India, Pakistan, Sweden, Kenya, Tanzania, Germany, Thailand, United Kingdom, Puerto Rico, Zambia, Canada, and Turkey) clustered with the Ethiopian accessions in SP1. Attempts to sub-cluster SP1 could not further differentiate the lines. The SP2 and AG groups consisted of the remaining lines from the Agrisoma collection and two accessions from Ethiopia.

Tree-based clustering and multi-dimensional scaling (MDS) approaches were utilized to support the STRUCTURE analysis. A neighbour-joining (NJ) tree was constructed using an un-weighted pair group method with arithmetic mean (UPGMA). The phylogeny confirmed that SP1 and SP2 separated into distinct clusters (Fig. 2b). In addition, SP1 was further grouped into three clusters, one major (72.2% accessions) and two minor (11.6% and 4.6% accessions). Further, principal component analysis (PCA) of the 620 accessions was concordant with both the STRUCTURE and phylogenetic analyses (Fig. 2c, Supplementary Table S3). The first five principal co-ordinates together account for only 13.8% of the variation present in the entire *B. carinata* collection (Supplementary Table S3).

Analysis of molecular variation was calculated to study genetic differentiation among STRUCTURE defined sub-populations. This analysis revealed low within sample (or individual) variance (14%) and between sub-population variance (23%), whereas a higher level of variance was observed within sub-populations (63%) (Supplementary Table S4). Pairwise *F*_*ST*_ was calculated to assess the significance of genetic differentiation between subpopulations and revealed a modest difference (0.148) between SP1 and SP2. In addition, *F*_*ST*_ estimates were 0.048 between SP1 and AG, and 0.082 between SP2 and AG. High fixation indices were observed (F_IS_ = 0.817; F_IT_ = 0.860, *P* ≤ 0.001) within each subpopulation, suggesting high levels of inbreeding. Relative kinship between *B. carinata* lines is depicted in Supplementary Fig. S1a; less than 40% of the lines (N = 247) had an observed kinship coefficient value ranging from 0.05 to 0.5 suggesting any kind of familial relatedness, notably these values were higher among the Agrisoma collection.

### Nucleotide diversity

Measures of nucleotide diversity for various genomic contexts are presented in Table 1 and Supplementary Figs. S2–S4. The estimates of nucleotide diversity across the whole genome and the two sub-genomes (Brassica B and C) were π = 1.31 × 10^–05^, 1.56 × 10^–05^, and 8.78 × 10^–06^, respectively, which were at least two-fold lower than those observed for *Brassica napus*^33,34^. Similarly, low estimates of Watterson’s θ were observed, whole genome = 6.60 × 10^–06^, B sub-genome = 7.83 × 10^–06^, and C sub-genome = 4.43 × 10^–07^, suggesting a low population mutation rate. Both of these measures highlight higher levels of nucleotide diversity across the B genome compared to the C. Considering the genome annotation, the level of nucleotide diversity was lowest in introns (8.61 × 10^–06^), followed by coding (9.23 × 10^–06^) and intergenic SNPs (1.00 × 10^−05^). There was no significant difference in the level of nucleotide diversity and Watterson’s estimator among structure defined subpopulations. The overall Tajima D distribution at the whole genome level (− 1.9 to 5.70) significantly deviated from neutrality, evidence of a recent selective sweep.

**Table 1:** Diversity statistics for various genomic contexts calculated over 100 kb non-overlapping windows across the *B. carinata* genome.

	Number of SNPs	Nucleotide diversity (π)^a^	Watterson’s θ^b^	Tajima’s D^c^
Total (BC genome)	10,199	1.31 × 10^–05^	6.60 × 10^–06^	1.30
B subgenome	7,452	1.56 × 10^–05^	7.83 × 10^–06^	1.35
C subgenome	2,747	8.78 × 10^–06^	4.43 × 10^–07^	1.21
Coding	4,475	9.23 × 10^–06^	4.68 × 10^–06^	1.23
Synonymous	2,988	7.72 × 10^–06^	3.93 × 10^–06^	1.16
Non-synonymous	1,487	6.46 × 10^–06^	3.30 × 10^–06^	1.12
Introns	2,435	8.61 × 10^–06^	4.40 × 10^–06^	1.19
Intergenic	3,183	1.10 × 10^–05^	4.85 × 10^–06^	1.09
SP1	9,575	1.36 × 10^–05^	6.60 × 10^–06^	1.43
SP2	4,604	1.71 × 10^–05^	8.89 × 10^–06^	1.43

^a^Nucleotide diversity (π); i.e., the average pairwise nucleotide differences per site.

^b^Waterson’s estimator of nucleotide diversity per site.

^c^Tajima’s D Neutrality test statistic.

### Genetic diversity

The pattern of genetic diversity among *B. carinata* accessions was evaluated using gene diversity (*H*_*E*_), observed heterozygosity (*H*_*O*_), and polymorphism information content (PIC) at individual genome (B and C), whole genome (BC) and subpopulation (SP1 and SP2) level. The *B. carinata* accessions showed a low level of *H*_*E*_ (0.30) and *H*_*O*_ (0.05) at the whole genome level (Supplementary Table S5). Between the B and C genomes, there was no significant difference for *H*_*E*_ and *H*_*O*_. Broadly, the observed heterozygosity was low compared to expected heterozygosity indicating inbreeding among *B. carinata* accessions. Within subpopulations, SP1 showed a similar level of *H*_*E*_ but higher *H*_*O*_ compared to SP2, implying more extensive inbreeding in the smaller subpopulation (Supplementary Table S5, Supplementary Fig. S1b). Although the number of polymorphic SNPs was higher in the B (73%) compared to the C genome (27%) (Supplementary Fig. S1c), PIC values were largely invariant across the genome (Supplementary Table S6). In general, the PIC values were largely mirrored in the two sub-populations with three notable exceptions: B3, B6 and C3 all showed lower PIC values in SP2 (Supplementary Table S6).

### Genome-wide patterns of linkage disequilibrium (LD)

**Figure 3:**
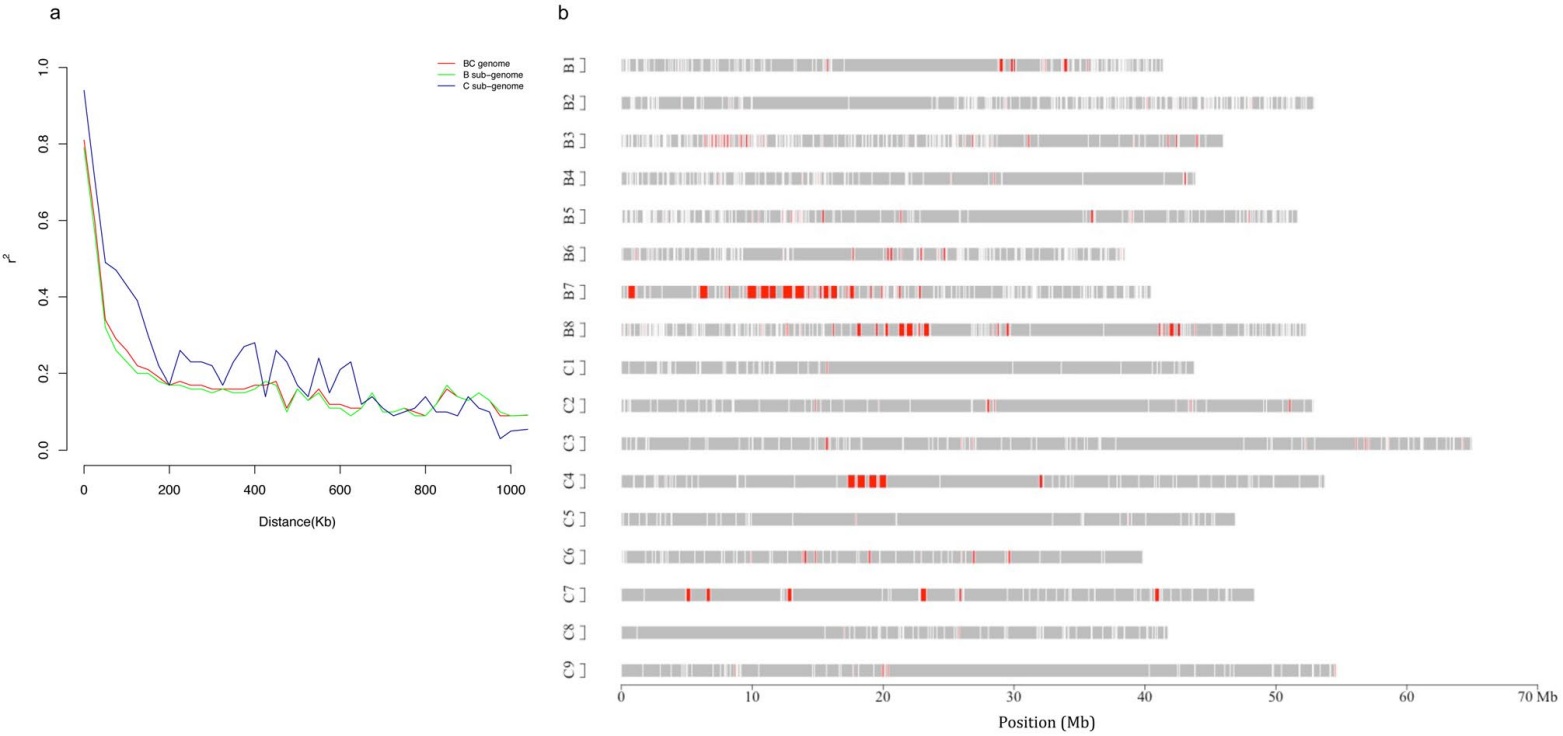
Linkage disequilibrium (LD) decay and genome-wide haplotype blocks. (**a**) LD decay at whole genome and subgenome level. Scatterplots showing *r*^2^ plotted against physical distance in kb. (**b**) Genome-wide distribution of haplotype blocks. Red rectangles represent genomic regions with haplotype blocks. Grey colours indicate genomic regions without haplotype blocks.

Pairwise *r*^2^ values were calculated using 7,452 and 2,747 polymorphic SNPs for the B and C genomes, respectively and 10,199 SNP for the composite BC genome (Table 2, Fig. 3). The *r*^2^ value was plotted against physical distance, and a critical value of *r*^2^ = 0.1 was used to estimate the extent of LD. The mean pairwise *r*^2^ value was estimated for the whole genome (0.077), B subgenome (0.076), and C subgenome (0.081). The mean genome-wide *r*^2^ values suggested high levels of LD in *B. carinata*, extending up to 700 kb in the whole genome (BC), and across 475 kb (excluding a co-inherited set of blocks on B3, see below) and 725 kb in Brassica B and C genomes, respectively (Fig. 3a). As recombination history is variable, different patterns of LD were observed in each chromosome ranging from 100 to 5,000 kb (Supplementary Fig. S5a–c). On average, LD extended over similar distances in the chromosomes of the two subgenomes, 357 and 322 kb in the B and C subgenomes, respectively. Within the B sub-genome, LD was high in B1 (350 kb), B3 (5,000 kb), B7 (775 kb), and B8 (525 kb), whereas in the C sub-genome, C2 (425 kb), C4 (675 kb) and C7 (525 kb) showed the highest LD. Different levels of LD decay were also detected in subpopulations (Supplementary Fig. S5d), where SP2 displayed extreme LD (> 50 Mb) compared to SP1 (475 kb), which was somewhat biased by the small number of closely related accessions in SP2.

**Table 2:** Linkage disequilibrium pattern and distribution of haplotype blocks in the *B. carinata* collection.

Chromosome	Number of SNPs	LD decay at *r*^2^ = 0.1 (kbp)	Mean *r*^2^	Number of haplotype blocks	Max size of block (kbp)	Recombination rate (ƿ/kb)
BC	10,199	700	0.077	1970	697.43	1.07
B	7,452	475	0.076	1,431	697.43	1.28
C	2,747	725	0.081	539	617.98	0.88
B1	771	350	0.073	146	284.77	1.47
B2	1,261	175	0.048	240	96.02	1.33
B3	1,207	5,000	0.121	227*	147.53	1.09
B4	599	200	0.057	118	155.05	1.73
B5	894	275	0.052	186	202.54	0.98
B6	809	200	0.056	165	190.35	1.22
B7	920	775	0.101	162	715.05	0.97
B8	991	525	0.061	188	506.46	1.50
C1	213	175	0.073	47	88.97	0.77
C2	266	425	0.108	51	188.85	0.73
C3	503	300	0.062	101	197.44	0.50
C4	300	675	0.170	56	617.98	0.53
C5	262	200	0.087	46	60.39	1.18
C6	270	300	0.066	57	188.77	0.65
C7	302	525	0.082	57	457.46	0.70
C8	348	100	0.062	66	68.43	1.61
C9	283	200	0.077	58	139.89	1.25
SP1	9,575	475	0.070	–	–	–
SP2	4,604	> 50,000	0.465	–	–	–

*Strong LD (FAE1 region) was excluded and LD recalculated for use in haplotype block analysis.

### Haplotype blocks

LD blocks, also known as haplotype blocks, were identified in *B. carinata* using the method described by Gabriel et al*.*^26^. A large set of adjacent blocks spanning 6.0 Mb was identified on B3, which confounded the genome-wide analyses; thus, the LD value used to estimate the block pattern for B3 excluded these blocks. The pattern of LD block distribution and total length varied significantly among the two sub-genomes, with higher numbers of blocks found in the Brassica B (1,431; 21.22 Mb) compared to the C genome (539; 7.95 Mb) (Table 2), likely as a result of the increased SNP density in the B genome. In line with this result, the percent frequency of larger haplotype blocks (> 51 kb) was slightly higher in the C (7.6%) than in the B sub-genome (7.3%) (Supplementary Fig. S6). The number and size of LD blocks varied greatly across each chromosome, apart from B3 a number of large haplotype blocks (> 400 kb) were observed on B7 (largest 715.05 kb), B8 (506.46 kb), C4 (617.98 kb) and C7 (457.46 kb) (Supplementary Table S7). Genome-wide scans using the pairwise *F*_*ST*_ statistics also identified the haplotype blocks on chromosome B3 and B8 as regions contributing to significant differentiation among the lines.

### Genomic regions under selection

A number of factors were utilised to identify genomic regions of interest (ROI) that appeared to be under selection within the *B. carinata* population; namely co-incidence of biased principal component loadings (Supplementary Fig. S7), localised high pairwise *F*_*ST*_ values, and the presence of haplotype blocks (Table 3). Utilising PC1 loadings, four regions were identified on three chromosomes, B1, B3, and C2 with blocks of SNPs with higher loading values (Supplementary Fig. S7). Of these, the ROI on B3, of about 4.87 Mbp, showed additional evidence of selection, being co-localized with a large haplotype block and a region of high *F*_*ST*_ (Supplementary Fig. S8a). PCA analyses based on the 620 samples using 294 SNPs from this region (0.62–1.1 Mb) clearly separated SP1 and SP2 (Supplementary Fig. S8b) with the top five PCs capturing over 80% of the observed variation, and the top two PCs 54% of the variation. PCA using SNPs from the other two potential ROI (B1 and C2) did not differentiate SP1 and SP2 but captured variation of 49 and 88%, respectively. An additional ROI was identified on B8, where a large haplotype block coincided with biased pairwise *F*_*ST*_ values.

**Table 3:** Genomic co-ordinates for regions of interest.

Chromosome	Coordinates (bp)	Length (Mbp)	Genes*	PC variation (%) (First two PCs)	
B1	36,151,527–37,051,510	0.90	*PHOSPHOENOLPYRUVATE CARBOXYLASE 3, HEME, EMBRYO DEFECTIVE 3,120* *LYSM RLK1-INTERACTING KINASE 1*	49.60	SP1 and SP2 are not differentiated
**B3**	**6,240,446–11,108,537**	**4.87**	*FAE1, CYP79B2, APK2, GSH1, IQD1, JAI1, DWARF2, HOMEOBOX PROTEIN16, VOZ1, EDF1*	**53.90**	**SP1 and SP2 are highly differentiated**
**B8**	**19,335,162–23,732,292**	**4.39**	*MAM1, AOP1, CYP79F1,BAT5, LIF2*	**75.20**	**SP1 and SP2 are differentiated**
C2-1	13,922,159–16,610,327	2.69	*LACS9, BCAT6, FLOWERING LOCUS T*	83.66	SP1 and SP2 are not differentiated
C2-2	27,814,654–29,475,511	1.66	*MAM1*	88.92	SP1 and SP2 are not differentiated

Bold values indicates multiple independent lines of evidence suggesting selection

*Candidates genes involved in fatty acid, glucosinolate and flowering pathway.

**Figure 4:**
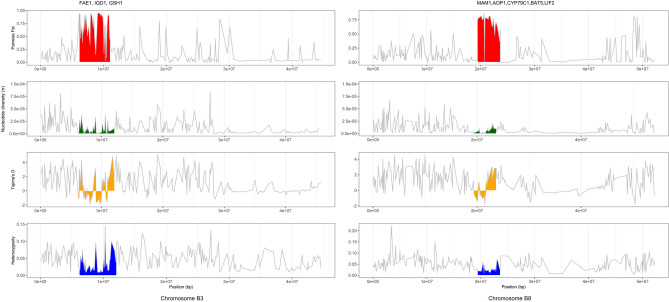
Recent selective sweep regions in *B. carinata* on Chromosomes B3 and B8. Each row from the top, calculated for all the SNPs with a non-overlapping a window of 100 kb, represents: level of genetic differentiation (Pairwise *F*_*ST*_); Nucleotide diversity estimates, Tajima’s D neutrality statistics; and Heterozygosity of SNPs.

Studying the genes annotated within the identified ROI, two of the regions suggested selection for seed quality traits (Table 3, Supplementary Table S8, S9). The region on B3 contained an orthologue of *fatty acid elongase1* and genes involved in the indole glucosinolate pathway, *IQD1* and *GSH1*; and the ROI on chromosome B8 (spanning 4.4 Mbp) harboured regulatory genes involved in biosynthesis and accumulation of aliphatic glucosinolates namely, *MAM1, AOP1, CYP79C1, BAT5*, and *LIF2* (Fig. 4).

## Discussion

A global collection of Ethiopian mustard was utilized to characterize molecular diversity, uncover population structure, and assess the potential of the available lines for GWAS. A large number of genome-wide SNPs were detected, which could be of value for future breeding efforts. *Brassica carinata* showed a low SNP frequency (one every 91.7 kb) and modest estimates of nucleotide diversity (π = 1.31 × 10^−05^) compared to its sister species *B. napus,* which ranged between π = 1.89 × 10^−3^ and 2.30 × 10^−3^ across sub-populations^34^. SNP frequency and nucleotide diversity are affected by several factors, including selection, mutation rates, breeding behaviour, and effective population size during species formation and demography^35^. The lower levels of nucleotide diversity suggest stronger genetic bottlenecks during domestication of *B. carinata* than for *B. napus*. Although the two species followed a similar evolutionary path, each likely formed from a limited number of hybridization events between progenitor diploid species leading to small effective population sizes; however, *B. napus* benefited from introgression with extant diploid species^36,37^.

The *B. carinata* population was clustered into two distinct groups (SP1 and SP2) based on breeding history rather than natural selection. Raman et al*.*^16^ observed a weak population structure among 83 *B. carinata* accessions with four STRUCTURE defined populations and the majority of the accessions (83.3%) in one group. Although not reflected in the current STRUCTURE analyses, a similar weak grouping effect was observed in the current data when analysed using tree-based approaches. The Bayesian clustering, phylogeny, and PCA analyses indicated that most accessions irrespective of their assigned country of origin were derived from Ethiopian accessions; in accordance with previous suggestions that Ethiopia is the primary centre of origin for *B. carinata* and the species has spread across different continents through migration with early human civilization. The only accessions that clustered based on origin other than Ethiopia was a small number of Zambian accessions. Similar results were reported in Teklewold and Becker^11^, suggesting either these accessions separated earlier from the centre of origin or there has been selection for locally adaptive traits, but this needs further investigation and ideally additional germplasm. There has been limited traditional breeding applied to *B. carinata* improvement, so perhaps not surprisingly the greatest impact to the observed population structure was found with the inclusion of accessions from a select Agrisoma collection, which separated from the Ethiopian lines forming the basis of SP2. Even with this differentiation, there is a low level of pairwise population differentiation between SP1 and SP2, since SP2 almost certainly shares a common origin with SP1 and selective breeding seems to have targeted particular regions of the genome. Similarly, analysis of molecular variance revealed low differentiation between sub-populations, suggesting high rates of gene flow between populations.

Recently formed species might be expected to have lower levels of genetic diversity^38^ as observed in recently formed polyploid crops, such as cotton^39^, peanut^40^, and soybean^41^. The limited natural allopolyploidization event(s) that likely resulted in the origin of *B. carinata* and its geographic isolation appears to have had a dramatic impact on the level of genetic diversity^42^. A low level of heterozygosity was found among *B. carinata* accessions, indicating adverse effects of the small population size or loss of heterozygosity due to genetic drift^43^. Lower values of observed versus expected heterozygosity suggest a high degree of inbreeding among *B. carinata* accessions could have resulted in fixation of alleles, which almost certainly contributed to lower genetic variation in the *B. carinata* genome.

The identification of polymorphic SNPs was significantly biased toward the B genome. Earlier studies similarly observed that the C subgenome is less polymorphic compared to both the A and B Brassica genomes, respectively^14,44^. There were marginally higher non-synonymous substitution rates observed in captured genic regions for the B sub-genome (52.5%) compared to the C sub-genome (41.6%) (Supplementary Table S2). The higher polymorphism rate in the Brassica B genome could be attributed to the earlier separation of *B. nigra* genome (8 Mya) than *B. oleracea* (4 Mya) from their shared common ancestor and thus could have accumulated higher levels of mutation^45^. It was noted that the effective recombination rate is lower across the C genome than the B genome (Table 2, Supplementary Fig. S9), although there did not appear to be a direct correlation at the individual chromosome level. In *B. napus* the lower polymorphism rate in the C genome compared to that in the A is often inferred to be due to recurrent introgression of *B. rapa* alleles during adaptation of the crop^46^; however, no such breeding history has been suggested for *B. carinata*. It is also possible that the higher mutation rate is indicative of C sub-genome dominance, leading to preferred maintenance of C genome orthologues after whole genome duplication^47^. The latter possibility would be intriguing since there has been no evidence of genome dominance found in the related allotetraploid *B. napus*^48^.

LD blocks or haplotype blocks indicate regions where limited recombination has led to co-inheritance of loci, potentially from the ancestral genome, largely as a consequence of selection and they have often been found to harbour domestication-related genes^26,49^. The power of genome-wide association mapping mainly depends on the rate of genome-wide LD and its distribution on different chromosomes^50^. *Brassica carinata* is a preferentially self-pollinating oilseed crop with limited outcrossing^51,52^, and thus a higher level of LD and lower degree of recombination might be expected. The present study demonstrated high LD (~ 700 kb) with a high mean pairwise *r*^2^ value (0.077) in *B. carinata*, consistent with previous work where an LD decay rate of ~ 525 kb was observed^17^. Also, similar rates of LD have been observed in closely related species, ~ 250 kb in *A. thaliana*^53^ and ~ 500 kb in *B. napus*^46^. Consistent with the lower rates of polymorphic loci the LD decay rate in the C sub-genome (~ 725 kb) was higher than the B sub-genome (~ 475 kb), which was comparable to a previous study, where the C sub-genome showed longer LD decay (~ 400 kb) than the B sub-genome (~ 250 kb)^17^. LD decay over the whole genome was used to predict the minimum number of SNPs required for genome-wide association studies in *B. carinata*. Considering LD at the whole genome level (700 kb) and a genome size of 1,284–1,544 Mbp, the number of SNPs required for successful implementation of LD mapping studies in *B. carinata* would be 1,834–2,206 SNPs. Nonetheless, higher LD will create resolution limits in mapping studies, thus a genome-wide association study may not yield strong positive signals due to an extensive haplotype pattern and the long range of LD in *B. carinata*^54^. There are various forces which affect the level of LD, including selection, tight linkage between genes, allele frequency, recombination rate, population size, and mating system^28^. In the studied lines the impact of small population size coupled with selection for specific traits in a breeding program led to the markedly higher level of LD observed in SP2 compared to that in SP1.

Two ROI were identified with multiple lines of evidence, including low nucleotide diversity, deviation from neutrality (Tajima’s D), biased PC loadings and pairwise *F*_*ST*_ values, which were suggestive of positive selection. The regions on B3 and B8 harboured well characterized fatty acid and glucosinolate biosynthesis genes, respectively, and both explained a significant amount of the variation between the SP1 and SP2 populations. These two regions bore all the hallmarks of selective breeding for seed quality traits, similar to observations made for selection of domestication traits^55,56^.

The present study provides a comprehensive analysis of diversity among *B. carinata* germplasm available from resource centres worldwide, and identified thousands of genome-wide SNPs using GBS*.* The diversity observed suggests *B. carinata* originated from a very limited number, if not a single hybridization event, with little or no subsequent inter-specific crossing with the parental progenitors. Although as observed in the Agrisoma collection, specific traits can be targeted for selection, further use of related diploid species may be necessary to increase the levels of available genetic diversity. The long-range LD and extended haplotype blocks could hamper fine resolution of trait loci that might be detected through GWAS. The best approach to crop improvement might be a joint linkage/association mapping approach such as nested association mapping, which can break long LD blocks into smaller fragments using recombination and establish greater genotype–phenotype correlations^25,57^.

## Materials and methods

### Plant material

A population of 631 *Brassica carinata* lines potentially representing 15 different countries were obtained from eight different gene banks, including Plant Gene Resources (PGRC) Canada (https://pgrc3.agr.gc.ca/index_e.html), Australian Temperate Field Crops Collection (ATFCC), Australia (https://www2.dpi.qld.gov.au/extra/asp/AusPGRIS/Centres.asp), LEIBNIZ-INSTITUT FÜR PFLANZENGENETIK UND KULTURPFLANZENFORSCHUNG (IPK) Germany (https://www.ipk-gatersleben.de/en/gbisipk-gaterslebendegbis-i/), Crop Research Institute (CRI) Czech Republic (https://www.vurv.cz/index.php?p=index&site=default_en ), National Center for Genetic Resources Preservation (NCGRP) USA (https://www.ars.usda.gov/main/site_main.htm?modecode=30-12-05-00), Warwick Genetic Resources Unit (WGRU) UK (https://www2.warwick.ac.uk/fac/sci/lifesci/wcc/gru/), Centre for Genetic Resources (CGN), The Netherlands (https://www.wageningenur.nl/en/Expertise-Services/Statutory-research-tasks/Centre-for-Genetic-Resources-the-Netherlands-1.htm), and Agrisoma (provided by Rick Bennett; https://agrisoma.com). Of the 631 accessions, 11 accessions were miss-identified *B. juncea* lines and were excluded from further analysis (Supplementary Table S1b).

### Genotyping by sequencing (GBS)

Genomic DNA of 3–4 week old seedlings was isolated using Qiagen DNeasy plant mini kit according to the manufacturer’s instructions (Qiagen Sciences, Maryland, USA). DNA was quantified using PicoGreen dsDNA assay kit (Invitrogen, Molecular Probes, Eugene, Orgon, USA) and the Victor X2 Fluorimeter (Perkin Elmer, Heidelberg, Germany) and then normalized to 20 ng/µl. GBS libraries were constructed according to Poland et al*.*^20^ using a double digestion approach with the enzymes *PstI* and *MspI*. Libraries were multiplexed at 96-fold and sequenced as 100 bp paired-end reads on an Illumina Hiseq2000. Sequence reads were aligned to the combined reference genomes of *B. nigra* (B, Ni100-SR)^31^ and *B. oleracea* (C)^32^using Bowtie2^58^. The parameters were set as: –local –sensitive –phred33 –minins 0 –maxins 1,000 –no-mixed –no-discordant –dovetail -k 50 –score-min L,0,0.8. Only unique matches to the genome were maintained for further analyses. SNPs were called using the genome analysis toolkit (GATK) version 3.2.2^59^. The parameters were as follows: -T UnifiedGenotyper, -glm BOTH, -ploidy 2. The missing SNP genotype calls were imputed using BEAGLE 4.0^60^ with the parameters as five initial burn-in iterations and ten iterations for estimating the genotype at missing data points. The imputation accuracy was assessed by altering known genotyping SNP calls as missing calls. The high-quality SNPs were named according to their base pair position within the diploid *B. nigra* and *B. oleracea* genome assemblies. Based on the genome annotation coordinates, the SNPs were categorised as coding, intergenic and intronic, the coding SNPs were further classified as synonymous or non-synonymous.

### Genetic analyses of population structure

Population structure analysis was performed based on 1,535 unlinked SNPs (*r*^2^ = 0.1) distributed on 17 chromosomes of *B. carinata* using STRUCTURE version 2.3.4 which employs a model-based clustering (Bayesian) approach^61^. The number of subgroups (K) was set from 1 to 10 using an admixture model with correlated allele frequency. For each K, five runs were performed separately using burn-in period of 100,000 iterations and 200,000 Markov Chain Monte Carlo repetitions (MCMC). The number of subpopulations were detected using an ad hoc statistic ∆K, the peak of ∆K value distribution, which is the rate of change of log probability of data between successive K values^62^. The probability of membership (Q value) equal or greater than 0.70 was taken as a threshold to assign genotypes to a particular subpopulation, those accessions with Q < 0.70 were retained as admixture. A neighbour joining (NJ) phylogenetic tree was performed with 10,000 bootstraps using DARwin 6.0.4^63^ and visualized using ITOL^64^. Principal component analysis (PCA) was performed using genome wide association and prediction integrated tool (GAPIT) package in R^65^.

### Analysis of genetic diversity

Genetic diversity parameters, including gene diversity (*D*), expected heterozygosity (*H*_*E*_), observed heterozygosity (*H*_*O*_), and polymorphism information content (PIC) of SNPs across sub-genomes and subpopulations were estimated using Powermarker software version 3.0^66^. Analysis of molecular variance (AMOVA) and pairwise *F*_*ST*_ was performed using GenAlex version 6.5^67^. The estimates of nucleotide diversity (average pairwise nucleotide differences (π)), Watterson Theta (θ_w_), and Tajima D were calculated using VariScan in 100 kb non-overlapping sliding windows^68,69^. To assess relatedness among genotypes, pairwise kinship analysis was performed using SPAGeDi v1.4^70^. Negative values between two individuals were changed to 0 as it indicates less relationship than expected between two random individuals^71^.

### Linkage disequilibrium (LD) estimation

The extent of LD across each chromosome was estimated using 10,199 high confidence SNPs. The pairwise *r*^2^ that represents the squared correlation coefficient between two SNP loci was calculated using TASSEL version 5.0 with a sliding window size of 500 kb^72^. The mean *r*^2^ values of significant pairs (*P* ≤ 0.05 in every 25 kb window) were plotted against physical distance to estimate the rate of LD decay for the whole genome, sub-genomes, and each chromosome separately. LD decay scatter plots were generated using R version 3 (R 3.4.0–3.6.0)^73^. LD blocks, also known as haplotype blocks, were identified using Haploview^74^, and the haplotype blocks were detected using all the SNPs by employing the block definition described in Gabriel et al.^26^. The *B. nigra* and *B. oleracea* genes residing in ≥ 200 kb of the LD blocks were identified, and putative functions were assigned based on those of their corresponding orthologues (best-BLAST-hit) in the related Brassicaceae *Arabidopsis thaliana*.

### Recombination rate

The genome-wide recombination rate rho (*ƿ*) was estimated on the subset of 96 representative accessions using the Interval program in LDhat 2.2^75^. In brief, 1,500,000 iterations were run, with sampling every 2000 iterations and a block penalty parameter of 5. The recombination rate was estimated for each chromosome separately and averaged across all the chromosomes to get an overall estimate.

### Region of interest

Principal component analysis was undertaken using GAPIT and the PC loadings were plotted across the chromosomes. The SNPs with high PC loadings (> 0.02) were identified as region of interest. The population differentiation statistics (Weir and Cockerhams’s Pairwise *F*_*ST*_) was estimated in an 100 kb sliding window using VCFTools^76^. To identify genes residing in an ROI, BLASTN analysis was performed against *Arabidopsis thaliana* (TAIR10) database using an E value of 1e−10^–6^.

## Supplementary information

Supplementary Information 1.

Supplementary Information 2.

Supplementary Information 3.

## References

[1] Jadhav A (2005). Increased levels of erucic acid in *Brassica carinata* by co-suppression and antisense repression of the endogenous FAD2 gene. Metab. Eng..

[2] Taylor DC (2010). *Brassica carinata*—a new molecular farming platform for delivering bio-industrial oil feedstocks: Case studies of genetic modifications to improve very long-chain fatty acid and oil content in seeds. Biofuel Bioprod. Biorefin..

[3] Nagaharu U (1935). Genome analysis in Brassica with special reference to the experimental formation of *B. napus* and peculiar mode of fertilization. Jpn. J. Bot..

[4] Johnston JS (2005). Evolution of genome size in *Brassicaceae*. Ann. Bot..

[5] Gómez-Campo, C. & Prakash, S. Origin and domestication. in *Developments in Plant Genetics and Breeding,* 33–58. (Elsevier, 1999).

[6] Ferreres, E., Fernandez, M., Minguez, I. & Dominguez, J. Productivity of *B. juncea* and *B. carinata* in relation to rapeseed. in *Proceedings of 6th International Rape-seed Congress, Paris, France,* 293–299 (1983).

[7] Getinet A, Rakow G, Downey R (1996). Agronomic performance and seed quality of Ethiopian mustard in Saskatchewan. Can J. Plant Sci..

[8] Gugel R, Seguin-Swartz G, Petrie G (1990). Pathogenicity of three isolates of *Leptosphaeria maculans* on Brassica species and other crucifers. Can. J. Plant Pathol..

[9] Yitbarek, S. Pathological research on noug, linseed, gomenzer and rapeseed in Ethiopia. in *First National Oilseeds Workshop, Addis Abeba (Ethiopia), 3–5 Dec 1991*. IAR (1992).

[10] Vicente G, Martínez M, Aracil J (2005). Optimization of *Brassica carinata* oil methanolysis for biodiesel production. J. Am. Oil Chem. Soc..

[11] Teklewold A, Becker HC (2006). Geographic pattern of genetic diversity among 43 Ethiopian mustard (*Brassica carinata* A. Braun) accessions as revealed by RAPD analysis. Genet. Resour. Crop Evol..

[12] Warwick S, Gugel R, McDonald T, Falk K (2006). Genetic variation of Ethiopian mustard (*Brassica carinata* A. Braun) germplasm in western Canada. Genet. Resour. Crop Evol..

[13] Guo S (2012). A genetic linkage map of *Brassica carinata* constructed with a doubled haploid population. Theor. Appl. Genet..

[14] Zou J (2014). Constructing a dense genetic linkage map and mapping QTL for the traits of flower development in *Brassica carinata*. Theor. Appl. Genet..

[15] Sharma BB, Kalia P, Yadava DK, Singh D, Sharma TR (2016). Genetics and molecular mapping of black rot resistance locus Xca1bc on chromosome B-7 in Ethiopian mustard (*Brassica carinata* A. Braun). PLoS ONE.

[16] Raman R (2017). Molecular diversity analysis and genetic mapping of pod shatter resistance loci in *Brassica carinata* L.. Front. Plant Sci..

[17] Zhang W (2017). Investigation of the genetic diversity and quantitative trait loci accounting for important agronomic and seed quality traits in *Brassica carinata*. Front. Plant Sci..

[18] He J (2014). Genotyping-by-sequencing (GBS), an ultimate marker-assisted selection (MAS) tool to accelerate plant breeding. Front. Plant Sci..

[19] Elshire RJ (2011). A robust, simple genotyping-by-sequencing (GBS) approach for high diversity species. PLoS ONE.

[20] Poland JA, Brown PJ, Sorrells ME, Jannink JL (2012). Development of high-density genetic maps for barley and wheat using a novel two-enzyme genotyping-by-sequencing approach. PLoS ONE.

[21] Huang Y-F, Poland JA, Wight CP, Jackson EW, Tinker NA (2014). Using genotyping-by-sequencing (GBS) for genomic discovery in cultivated oat. PLoS ONE.

[22] Lin M (2015). Genotyping-by-sequencing (GBS) identified SNP tightly linked to QTL for pre-harvest sprouting resistance. Theor. Appl. Genet..

[23] Alipour H (2017). Genotyping-by-sequencing (GBS) revealed molecular genetic diversity of Iranian wheat landraces and cultivars. Front. Plant Sci..

[24] Wang J, Street NR, Scofield DG, Ingvarsson PK (2016). Natural selection and recombination rate variation shape nucleotide polymorphism across the genomes of three related Populus species. Genetics.

[25] Myles S (2009). Association mapping: Critical considerations shift from genotyping to experimental design. Plant Cell.

[26] Gabriel SB (2002). The structure of haplotype blocks in the human genome. Science.

[27] Pavlidis P, Alachiotis N (2017). A survey of methods and tools to detect recent and strong positive selection. J. Biol. Res. (Thessalon).

[28] Flint-Garcia SA, Thornsberry JM, Buckler ES (2003). Structure of linkage disequilibrium in plants. Annu. Rev. Plant Biol..

[29] Zhu C, Gore M, Buckler ES, Yu JJ (2008). Status and prospects of association mapping in plants. Plant Genome.

[30] Falk, K.C. Development of early maturing *Brassica carinata* for western Canada. in *10th International Rapeseed Congress,* 26–29 (1999).

[31] Perumal S (2020). High contiguity long read assembly of *Brassica nigra* allows localization of active centromeres and provides insights into the ancestral *Brassica* genome. JAMA.

[32] Parkin IA (2014). Transcriptome and methylome profiling reveals relics of genome dominance in the mesopolyploid *Brassica oleracea*. Genome Biol..

[33] Nei M, Li WH (1979). Mathematical model for studying genetic variation in terms of restriction endonucleases. Proc. Natl. Acad. Sci. U.S.A..

[34] Gazave E (2016). Population genomic analysis reveals differential evolutionary histories and patterns of diversity across subgenomes and subpopulations of *Brassica napus* L.. Front. Plant Sci..

[35] Nei M (1987). Molecular Evolutionary Genetics.

[36] Dhaliwal I (2017). Cytogenetic and molecular characterization of B-genome introgression lines of *Brassica napus* L.. G3 Genes. Genom. Genet..

[37] Hu D (2019). Reconstituting the genome of a young allopolyploid crop, *Brassica napus*, with its related species. Plant Biotechnol. J..

[38] Pannell JR, Dorken ME (2006). Colonisation as a common denominator in plant metapopulations and range expansions: Effects on genetic diversity and sexual systems. Landsc. Ecol..

[39] Van Esbroeck GA, Bowman DT, Calhoun DS, May OL (1998). Changes in the genetic diversity of cotton in the USA from 1970 to 1995. Crop Sci..

[40] de Carvalho M (2004). Genetic diversity of peanut (*Arachis hypogaea* L.) and its wild relatives based on the analysis of hypervariable regions of the genome. BMC Plant Biol..

[41] Hyten DL (2006). Impacts of genetic bottlenecks on soybean genome diversity. Proc. Natl. Acad. Sci. U.S.A..

[42] Dixon GR (2007). Vegetable Brassicas and Related Crucifers.

[43] Wahlund S (1928). The combination of populations and the appearance of correlation examined from the standpoint of the study of heredity. Hereditas.

[44] Bancroft I (2011). Dissecting the genome of the polyploid crop oilseed rape by transcriptome sequencing. Nat. Biotechnol..

[45] Lysak MA, Koch MA, Pecinka A, Schubert I (2005). Chromosome triplication found across the tribe *Brassiceae*. Genome Res..

[46] Delourme R (2013). High-density SNP-based genetic map development and linkage disequilibrium assessment in *Brassica napus* L.. BMC Genom..

[47] Schnable JC, Springer NM, Freeling M (2011). Differentiation of the maize subgenomes by genome dominance and both ancient and ongoing gene loss. Proc. Natl. Acad. Sci. U.S.A..

[48] Chalhoub B (2014). Early allopolyploid evolution in the post-Neolithic *Brassica napus* oilseed genome. Science.

[49] Takano-Kai N (2009). Evolutionary history of GS3, a gene conferring grain length in rice. Genetics.

[50] Sorrells ME, Yu J, Feuillet C, Muehlbauer GJ (2009). Linkage disequilibrium and association mapping in the Triticeae. Genetics and Genomics of the Triticeae.

[51] Velasco, L. & Fernández-Martínez, J.M. Other brassicas. in *Oil Crops,* 127–153 (Springer, 2009).

[52] Cheung KW, Razeq FM, Sauder CA, James T, Martin SL (2015). Bidirectional but asymmetrical sexual hybridization between *Brassica carinata* and *Sinapis arvensis* (*Brassicaceae*). J. Plant Res..

[53] Nordborg M (2002). The extent of linkage disequilibrium in *Arabidopsis thaliana*. Nat. Genet..

[54] Larsson SJ, Lipka AE, Buckler ES (2013). Lessons from Dwarf8 on the strengths and weaknesses of structured association mapping. PLoS Genet..

[55] Huang X (2012). A map of rice genome variation reveals the origin of cultivated rice. Nature.

[56] Zhou Z (2015). Resequencing 302 wild and cultivated accessions identifies genes related to domestication and improvement in soybean. Nat. Biotechnol..

[57] Wu R, Zeng ZB (2001). Joint linkage and linkage disequilibrium mapping in natural populations. Genetics.

[58] Langmead B, Salzberg SL (2012). Fast gapped-read alignment with Bowtie 2. Nat. Methods.

[59] McKenna A (2010). The genome analysis toolkit: A MapReduce framework for analyzing next-generation DNA sequencing data. Genome Res..

[60] Browning SR, Browning BL (2007). Rapid and accurate haplotype phasing and missing-data inference for whole-genome association studies by use of localized haplotype clustering. Am. J. Hum. Genet..

[61] Pritchard JK, Stephens M, Donnelly P (2000). Inference of population structure using multi-locus genotype data. Genetics.

[62] Evanno G, Regnaut S, Goudet J (2005). Detecting the number of clusters of individuals using the software STRUCTURE: A simulation study. Mol. Ecol..

[63] Perrier, X. & Jacquemoud-Collet, J. DARwin software: Dissimilarity analysis and representation for windows (2006).

[64] Letunic I, Bork P (2016). Interactive tree of life (iTOL) v3: An online tool for the display and annotation of phylogenetic and other trees. Nucleic Acids Res..

[65] Lipka AE (2012). GAPIT: Genome association and prediction integrated tool. Bioinformatics.

[66] Liu K, Muse SV (2005). PowerMarker: An integrated analysis environment for genetic marker analysis. Bioinformatics.

[67] Peakall R, Smouse PE (2006). GENALEX 6: Genetic analysis in Excel. Population genetic software for teaching and research. Mol. Ecol. Notes.

[68] Vilella AJ, Blanco-Garcia A, Hutter S, Rozas J (2005). VariScan: Analysis of evolutionary patterns from large-scale DNA sequence polymorphism data. Bioinformatics.

[69] Hutter S, Vilella AJ, Rozas J (2006). Genome-wide DNA polymorphism analyses using VariScan. BMC Bioinform..

[70] Hardy OJ, Vekemans X (2002). SPAGeDi: A versatile computer program to analyse spatial genetic structure at the individual or population levels. Mol. Ecol. Notes.

[71] Yu J (2006). A unified mixed-model method for association mapping that accounts for multiple levels of relatedness. Nat. Genet..

[72] Bradbury PJ (2007). TASSEL: Software for association mapping of complex traits in diverse samples. Bioinformatics.

[73] R Development Core Team. R: A language and environment for statistical computing. R version 3 (R 3.4.0–3.6.0). R Foundation for Statistical Computing, Vienna. (Accessed 02 July 2018); https://www.R-project.org (2011).

[74] Barrett JC, Fry B, Maller J, Daly MJ (2004). Haploview: Analysis and visualization of LD and haplotype maps. Bioinformatics.

[75] Auton A, McVean G (2007). Recombination rate estimation in the presence of hotspots. Genome Res..

[76] Danecek P (2011). The variant call format and VCFtools. Bioinformatics.

