# Supplementary material for: Narrow genetic base shapes population structure and linkage disequilibrium in an industrial oilseed crop, Brassica carinata A. Braun
Source: Sci Rep. 2020 Jul 28;10:12629. doi: 10.1038/s41598-020-69255-w (PMC7387349; doi:10.1038/s41598-020-69255-w)
Supplement: Supplementary file 1 — Supplementary Information 1. [file 41598_2020_69255_MOESM1_ESM.pdf]

Title: Narrow genetic base shapes population structure and linkage disequilibrium in an industrial oilseed crop, Brassica carinata A. Braun

Authors: Yogendra Khedikar, Wayne E. Clarke, Lifeng Chen, Erin E. Higgins, Sateesh Kagale, Chu Shin Koh, Rick Bennett, Isobel A. P. Parkin*

Figure S1. Genetic diversity analysis in *B. carinata*. a) The distribution of relative kinship values among 620 carinata lines. Note: Values equal or greater than 0.5 were grouped as 0.5. (b) Estimate of heterozygosity in SP1, SP2 and AG. (c) Polymorphism information content (PIC) of 10,199 SNPs in the B. carinata.
Figure S2. The measures of genetic differentiation (Pairwise FST) with a window size of 100 kb across 19 chromosomes of *B. carinata*.

Figure S3. Nucleotide diversity estimates with a window size of 100 kb across 19 chromosomes of *B. carinata*.

Figure S4. Tajima's D neutrality test statistics with a window size of 100 kb across 19 chromosomes of *B. carinata*.

Figure S5. Linkage disequilibrium (LD) decay estimated using the *B. carinata* B, C subgenomes and subpopulations identified in structure analysis. (a) LD decay on each chromosome of B subgenomes and (b) LD decay on each chromosome of C subgenomes (c) LD decay on each chromosome of C subgenome after removing strong LD regions in C2 and C4 (d) LD decay on STRUCTURE identified sub-populations (SP1 and SP2). Scatterplots showing r2 plotted against physical distance in kbp.

Figure S6. Percent frequency of haplotype blocks in B and C subgenomes.

Figure S7. Principal Component loadings for SNPs on different *B. carinata* chromosomes. The PC1 loading plotted against physical distance in Mb.

Figure S8. The Region of Interest (ROI) on B3. a) A major haplotype block and the region of interest on B3 b) Principal component analysis showing higher differentiation for SP1 and SP2, the first two PCs capturing 54% variation.

Figure S9. Distribution of recombination rate as rho/kb across different chromosomes of *B. carinata*.

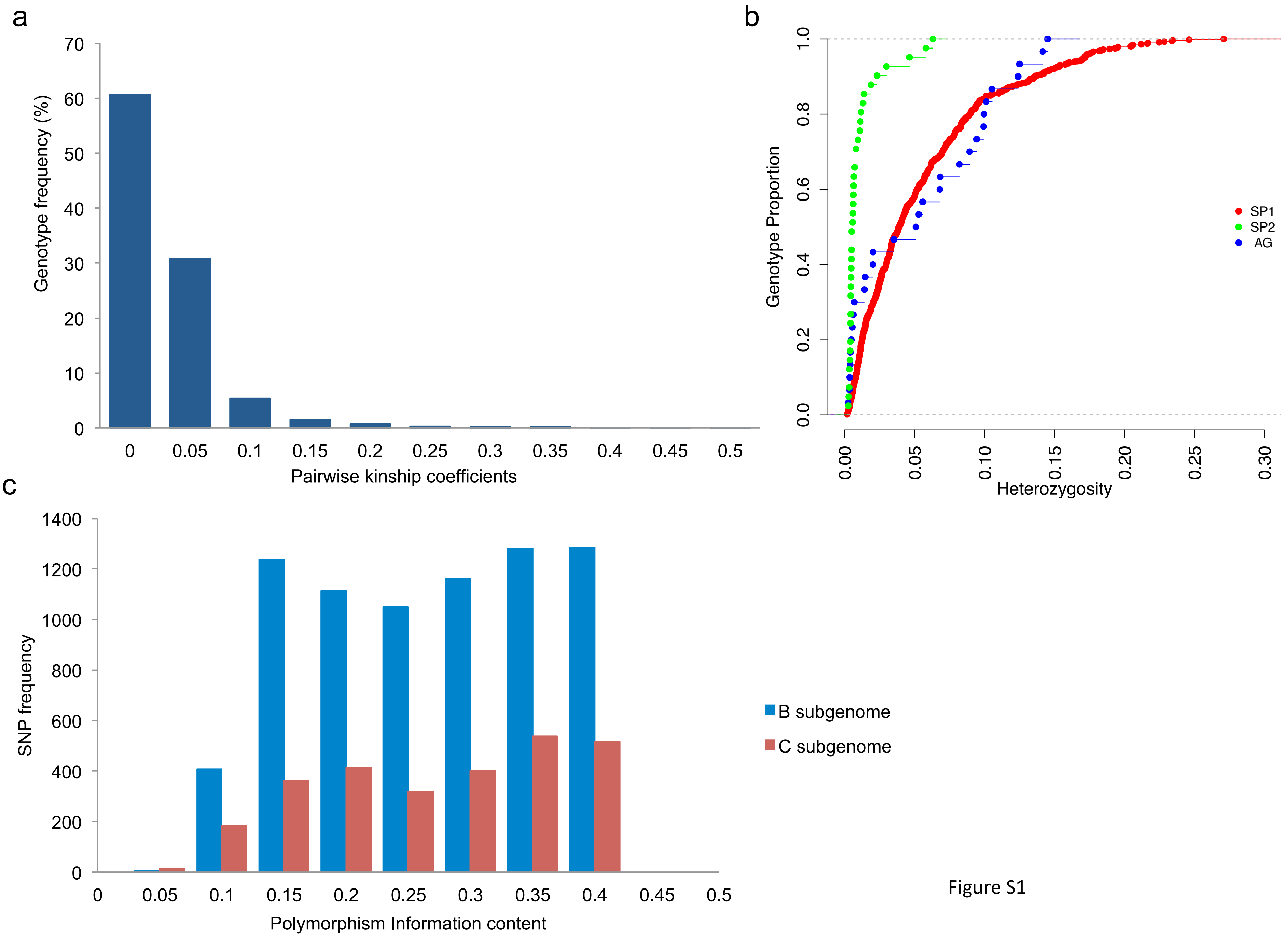
a
Genotype frequency (%)
70
60
50
40
30
20
10
0
0
0.05
0.1
0.15
0.2
0.25
0.3
0.35
0.4
0.45
0.5
Pairwise kinship coefficients
b
Genotype Proportion
1.0
0.8
0.6
0.4
0.2
0.0
0.00
0.05
0.10
0.15
0.20
0.25
0.30
Heterozygosity
SP1
SP2
AG
c
SNP frequency
1400
1200
1000
800
600
400
200
0
0
0.05
0.1
0.15
0.2
0.25
0.3
0.35
0.4
0.45
0.5
Polymorphism Information content
B subgenome
C subgenome

Figure S1

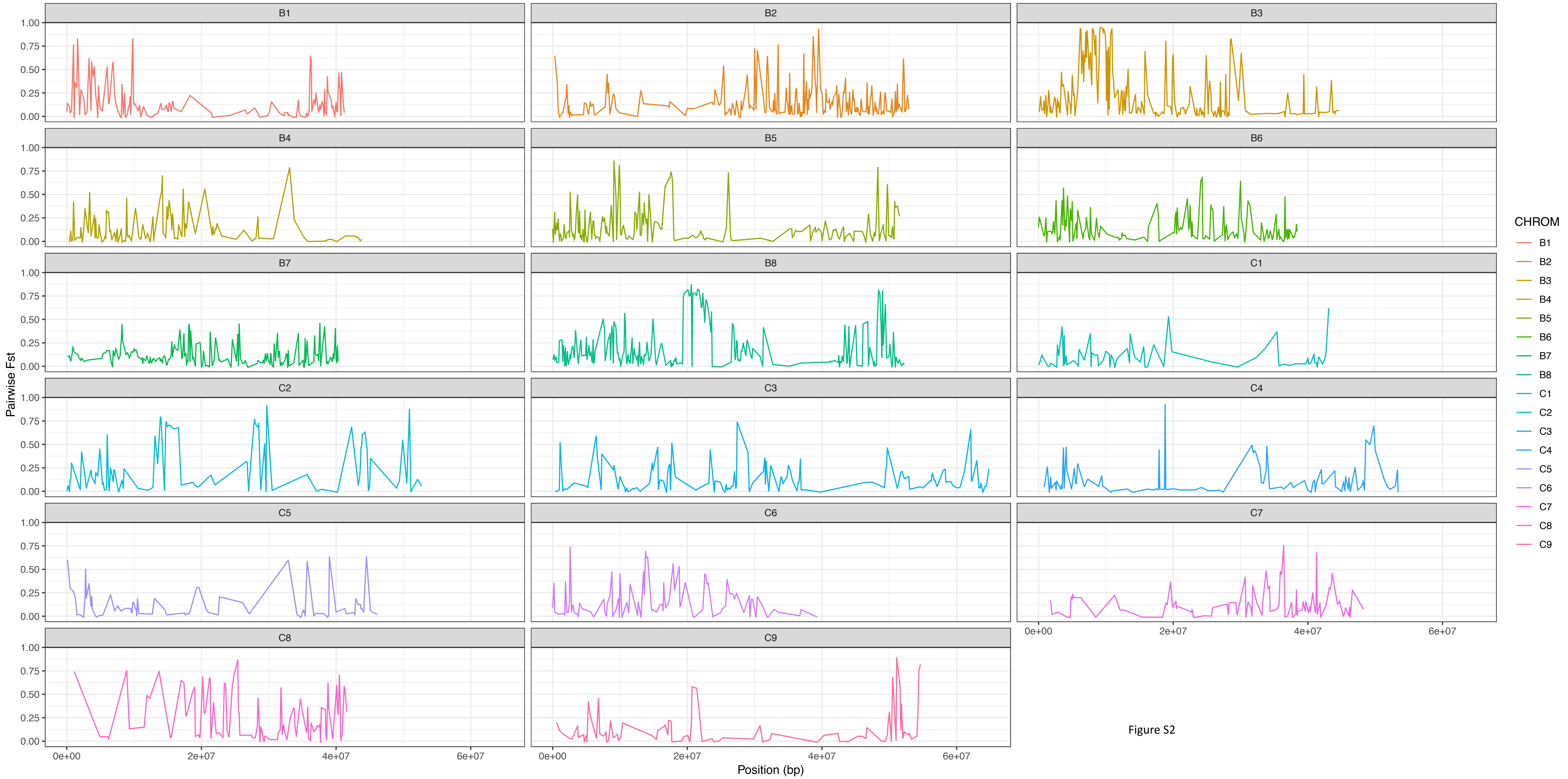

B1
B2
B3
B4
B5
B6
B7
B8
C1
C2
C3
C4
C5
C6
C7
C8
C9
Pairwise Fst
Position (bp)
0e+00
2e+07
4e+07
6e+07
0.00
0.25
0.50
0.75
1.00
CHROM

Figure S2

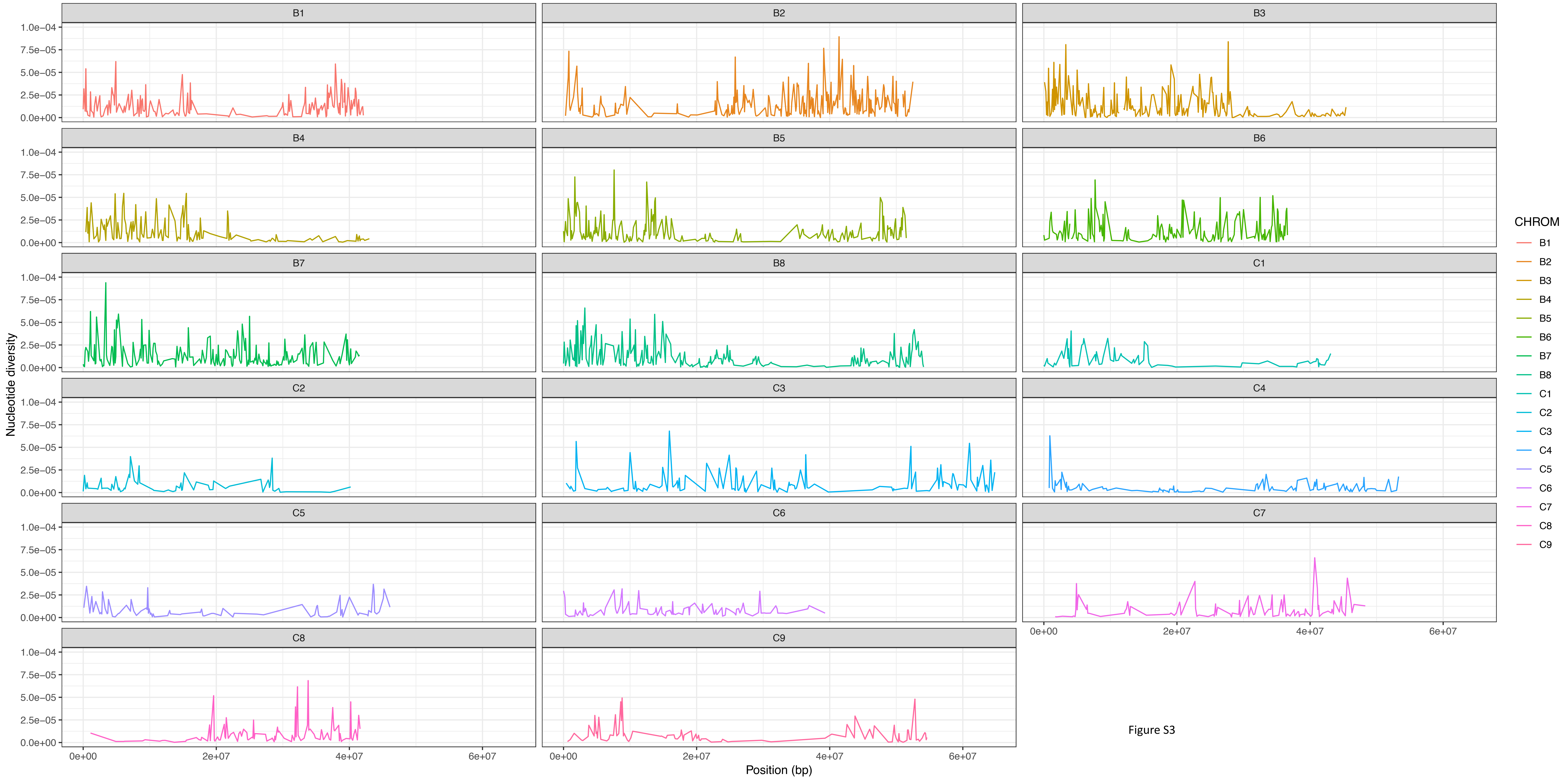
B1
B2
B3
B4
B5
B6
B7
B8
C1
C2
C3
C4
C5
C6
C7
C8
C9
1.0e−04
7.5e−05
5.0e−05
2.5e−05
0.0e+00
Nucleotide diversity
0e+00
2e+07
4e+07
6e+07
Position (bp)
CHROM
B1
B2
B3
B4
B5
B6
B7
B8
C1
C2
C3
C4
C5
C6
C7
C8
C9

Figure S3

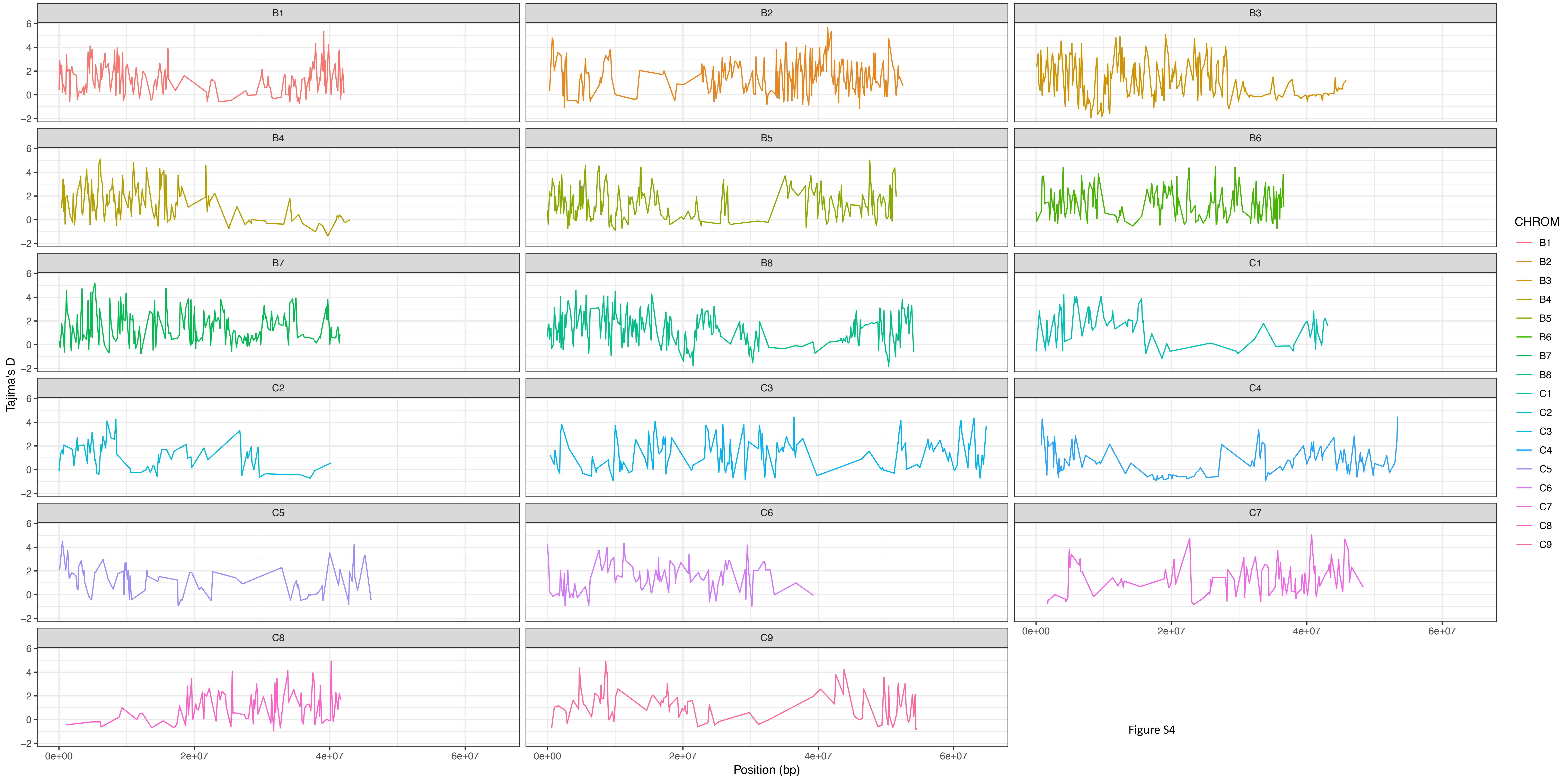
B1
B2
B3
B4
B5
B6
B7
B8
C1
C2
C3
C4
C5
C6
C7
C8
C9
Tajima's D
Position (bp)
0e+00
2e+07
4e+07
6e+07
CHROM

Figure S4

Figure S5

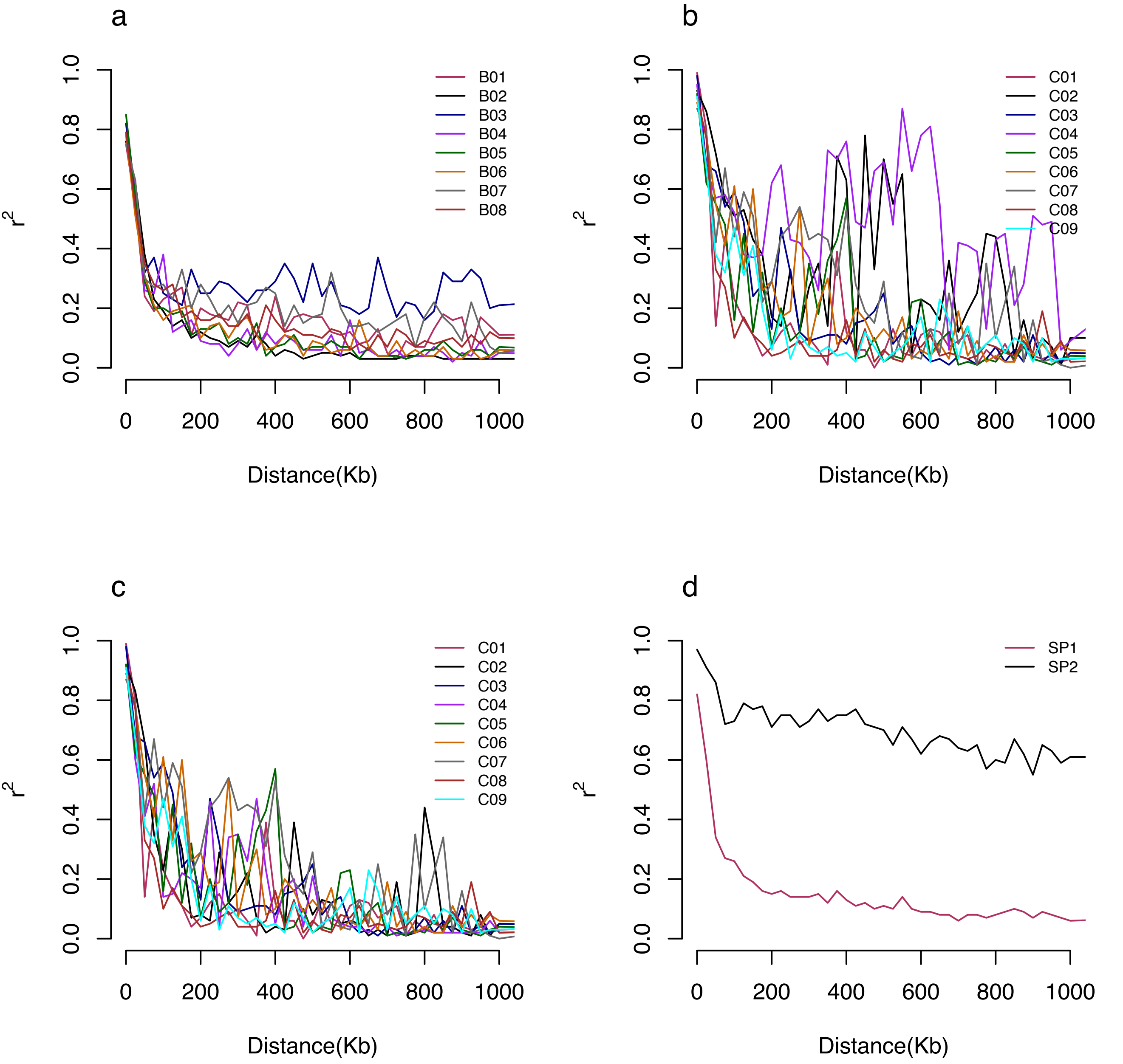
a
b
c
d
r2
Distance(Kb)
0.0
0.2
0.4
0.6
0.8
1.0
0
200
400
600
800
1000
B01
B02
B03
B04
B05
B06
B07
B08
C01
C02
C03
C04
C05
C06
C07
C08
C09
SP1
SP2

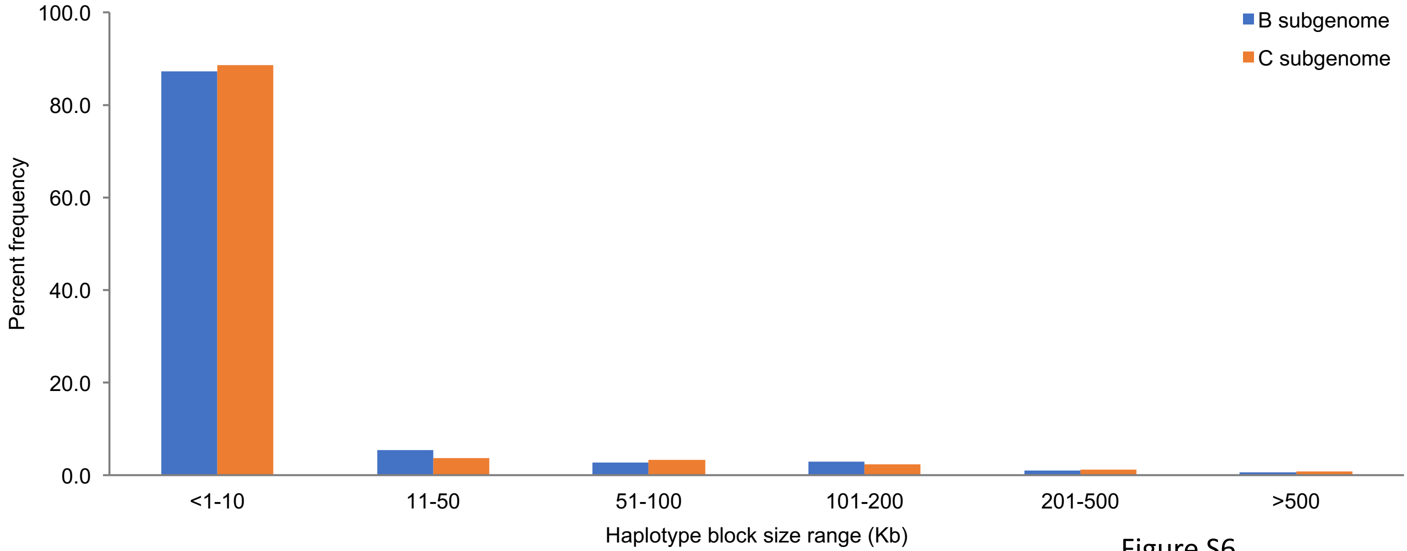

100.0
80.0
60.0
40.0
20.0
0.0
Percent frequency
B subgenome
C subgenome
<1-10
11-50
51-100
101-200
201-500
>500
Haplotype block size range (Kb)

Figure S6

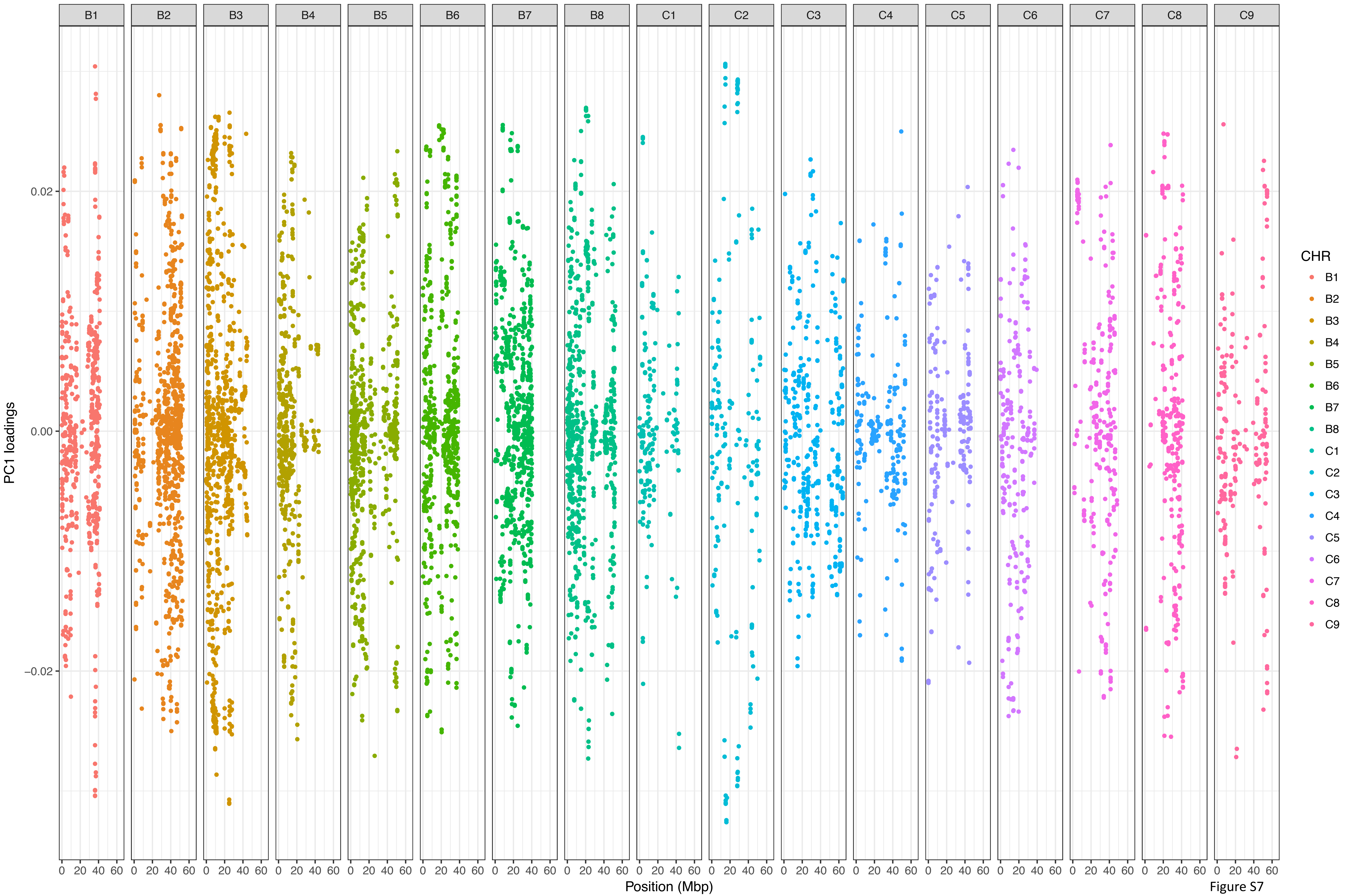
B1
B2
B3
B4
B5
B6
B7
B8
C1
C2
C3
C4
C5
C6
C7
C8
C9
PC1 loadings
0.02
0.00
-0.02
0 20 40 60
Position (Mbp)
CHR
B1
B2
B3
B4
B5
B6
B7
B8
C1
C2
C3
C4
C5
C6
C7
C8
C9

Figure S7

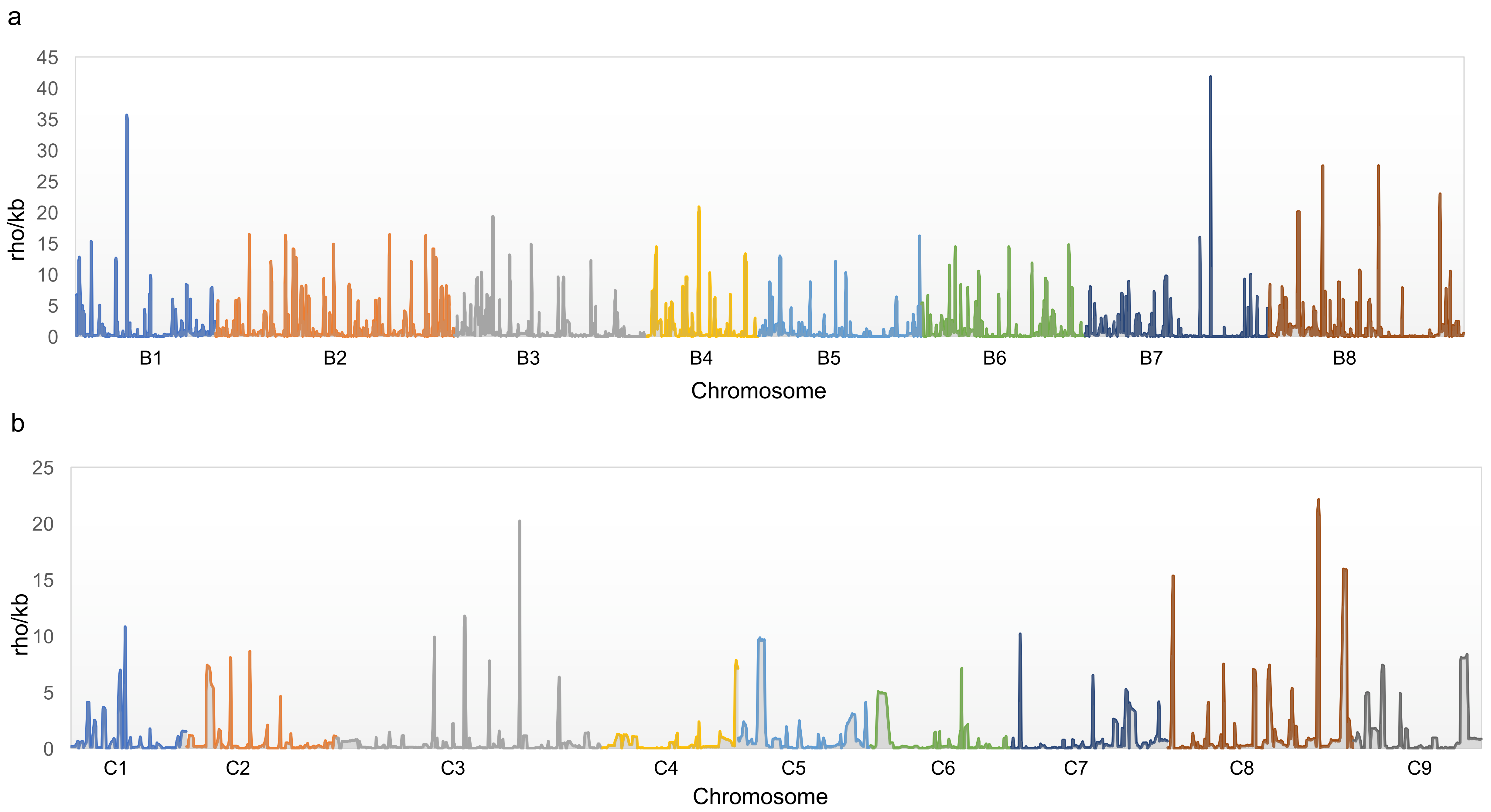
a
45
40
35
30
25
20
15
10
5
0
rho/kb
B1
B2
B3
B4
B5
B6
B7
B8
Chromosome
b
25
20
15
10
5
0
rho/kb
C1
C2
C3
C4
C5
C6
C7
C8
C9
Chromosome

Figure S8

a

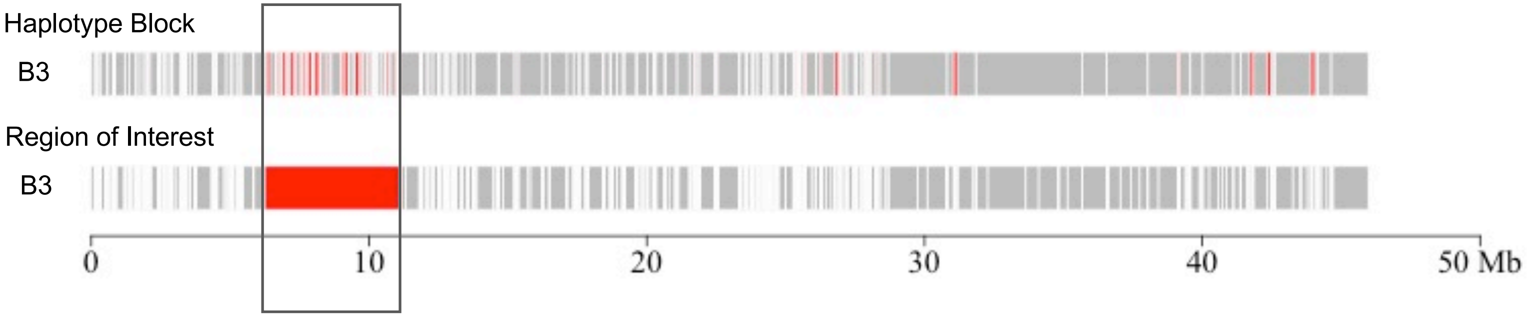

Haplotype Block
B3
Region of Interest
B3
0
10
20
30
40
50 Mb

b

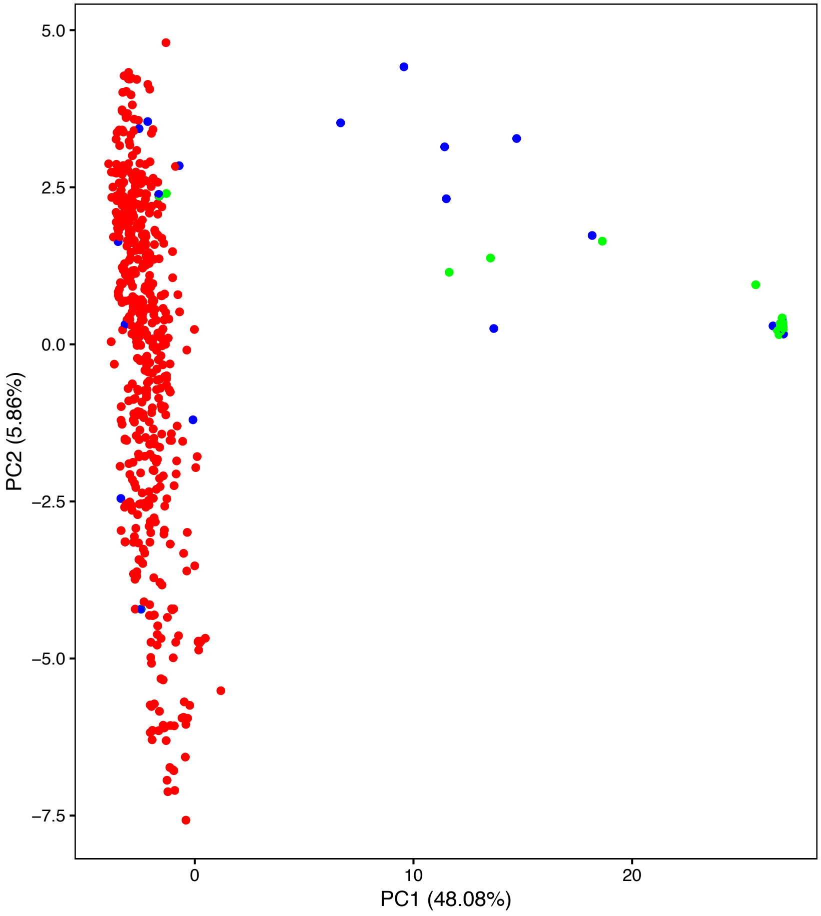

5.0
2.5
0.0
−2.5
−5.0
−7.5
PC2 (5.86%)
0
10
20
PC1 (48.08%)

Figure S9
